# Systemic inflammation affects reperfusion following transient cerebral ischaemia

**DOI:** 10.1016/j.expneurol.2016.01.013

**Published:** 2016-03

**Authors:** F. Burrows, M.J. Haley, E. Scott, G. Coutts, C.B. Lawrence, S.M. Allan, I. Schiessl

**Affiliations:** Faculty of Life Sciences, The University of Manchester, Stopford Building, Oxford Road, M13 9PT Manchester, UK

**Keywords:** Stroke, Co-morbidity, Systemic inflammation, Middle cerebral artery occlusion, Spectroscopy, Optical imaging, Reperfusion, Interleukin-1, Interleukin-1β

## Abstract

Reperfusion after stroke is critical for improved patient survival and recovery and can be achieved clinically through pharmacological (recombinant tissue plasminogen activator) or physical (endovascular intervention) means. Yet these approaches remain confined to a small percentage of stroke patients, often with incomplete reperfusion, and therefore there is an urgent need to learn more about the mechanisms underlying the no-reflow phenomenon that prevents restoration of adequate microvascular perfusion. Recent evidence suggests systemic inflammation as an important contributor to no-reflow and to further investigate this here we inject interleukin 1 (IL-1) i.p. 30 min prior to an ischaemic challenge using a remote filament to occlude the middle cerebral artery (MCA) in mice. Before, during and after the injection of IL-1 and occlusion we use two-dimensional optical imaging spectroscopy to record the spatial and temporal dynamics of oxyhaemoglobin concentration in the cortical areas supplied by the MCA. Our results reveal that systemic inflammation significantly reduces oxyhaemoglobin reperfusion as early as 3 h after filament removal compared to vehicle injected animals. CD41 immunohistochemistry shows a significant increase of hyper-coagulated platelets within the microvessels in the stroked cortex of the IL-1 group compared to vehicle. We also observed an increase of pathophysiological biomarkers of ischaemic damage including elevated microglial activation co-localized with interleukin 1α (IL-1α), increased blood brain barrier breakdown as shown by IgG infiltration and increased pyknotic morphological changes of cresyl violet stained neurons. These data confirm systemic inflammation as an underlying cause of no-reflow in the post-ischaemic brain and that appropriate anti-inflammatory approaches could be beneficial in treating ischaemic stroke.

## Highlights

• Systemic inflammation significantly increases reperfusion deficits as early as 180 min after stroke compared to vehicle.
• Platelet aggregates were found in the capillaries suggesting a key role in reducing blood flow through mechanical obstruction.
• IL-1 induced inflammation significantly increases the loss of neurons and leads to morphological changes 6 h after MCAo.

## 1 Introduction

Stroke is a leading cause of global morbidity and mortality with treatment options limited to reperfusion through thrombolysis or thrombectomy. Ischaemic stroke accounts for 85% of all human strokes and is mainly caused by occlusion of the middle cerebral artery (MCA), a major arterial branch supplying blood to the brain (Doyle et al., 2008, Lloyd, 2010, Rosamond et al., 2008). The resulting occlusion results in hypoperfusion of brain tissue creating an energy depleted state within the infarct core, triggering acute pathophysiological processes which result in neuronal injury (Ankolekar et al., 2012, Cramer et al., 2003, Semenza, 2009). Hypoperfusion starves tissue of glucose resulting in neuronal dysfunction and release of pro-inflammatory cytokines (Mori et al., 1992, Vila et al., 2000). Future successful treatment of ischaemic stroke requires a comprehensive understanding of the pathophysiological changes that occur within the acute phase of cerebral ischaemia.

Despite promising results in experimental studies of stroke in animal models there has been a lack of translation to clinical success (Ankolekar et al., 2012, Denes et al., 2010a, Denes et al., 2010b). Pre-clinical studies of ischaemic stroke to date have largely failed to take into account relevant co-morbidities for stroke, which experimentally we have shown can dramatically exacerbate ischaemic injury (Denes et al., 2010a, Denes et al., 2010b, McColl et al., 2007a, McColl et al., 2007b, McColl, 2008). A key hallmark of stroke co-morbidities is systemic inflammation and it is now well accepted that inflammatory processes are a major contributor to cerebral ischaemia (Denes et al., 2010a, Denes et al., 2010b, McColl et al., 2007a, McColl et al., 2007b, McColl, 2009, McColl, 2008, McColl et al., 2010, Maysami et al., 2015). The pro-inflammatory cytokine interleukin-1 (IL-1) in particular has been identified as a key mediator of neuronal injury. Experimental models of stroke have found that IL-1 signalling on endothelial cells, microglia, astrocytes and neutrophils stabilizes mRNA for pro-inflammatory mediator expression (Brandolini et al., 1997, Ericsson et al., 1995). Furthermore IL-1 acts on cerebral microvasculature endothelial cells, upregulating adhesion molecule expression and subsequent neutrophil transmigration (Brandolini et al., 1997). IL-1 has also been shown as the main driver in lipopolysaccharide (LPS) mediated worsening of damage (Denes et al., 2010a, Denes et al., 2010b, McColl et al., 2007a, McColl et al., 2007b). Recent findings demonstrate that systemic IL-1 prevents microvascular reperfusion post-stroke through endothelin-dependent mechanisms (Murray et al., 2013). Furthermore we have previously shown that preceding pneumonia infection worsens stroke outcome via IL-1 and platelet dependent mechanisms (Denes et al., 2014). Patients with underlying inflammation-related co-morbidities for stroke, including obesity, diabetes, arthritis and smoking often have worse outcomes following a cerebral ischaemic event (Bottcher and Falk, 1999, Herz et al., 2015, Pinto et al., 2004). In human trials IL-1 receptor antagonist (IL-1RA) limits the action of IL-1, improving patient outcome by reducing the pathophysiological activity including reduction in neutrophil numbers (Emsley et al., 2005, Emsley et al., 2008, Smith et al., 2004). Therefore we have identified IL-1 as a very relevant cytokine to co-administer with cerebral ischaemia. Despite these data supporting a key role of IL-1 in ischaemic brain injury it remains to be fully defined how IL-1 affects reperfusion post-stroke. To investigate this further we combine systemic administration of IL-1 with a remote filament model of middle cerebral artery occlusion (MCAo) (Burrows et al., 2015), using two-dimensional optical imaging spectroscopy (2D-OIS), to confirm the effects of IL-1 on cortical oxyhaemoglobin dynamics during reperfusion after stroke in mice.

## 2 Methods

### 2.1 Animals

All animal procedures were performed under an appropriate Home Office Licence and adhered to regulations as specified in the Animals (Scientific Procedures) Act (1986). Studies were performed in accordance with ARRIVE guidelines, with appropriate randomisation and blinding procedures in place, and ethical approval by the local ethics committee of the University of Manchester. Mice were kept at 21 °C and 65% humidity with a regulated 12-h light–dark cycle and free access to food and water. Twenty-nine male C57/BL6 mice (Harlan Laboratories, UK) weighing between 30 and 36 g (age 18 to 25 weeks) were used in this study. Ten C57/BL6 male mice underwent i.p. injection of 20 ng/kg mouse recombinant IL-1β (R&D systems, UK) in sterile PBS and 0.5% low endotoxin BSA, 30 min prior to a 30 min MCAo with 6 h reperfusion; the remaining ten mice underwent i.p. injection of sterile PBS with 0.5% low endotoxin BSA alone (vehicle), 30 min prior to a 30 min ischaemic insult with 6 h reperfusion. In addition four C57/BL6 mice underwent the IL-1 protocol and five C57/BL6 mice underwent the vehicle protocol but were sacrificed 10 min after removal of the MCAo filament (i.e. 70 min after injection) for further immunohistochemical analysis. Though IL-1β was injected in the interest of brevity the rest of the manuscript will simply refer to IL-1.

### 2.2 Remote filaments

Filament preparation was performed as described in (Burrows et al., 2015) producing 5 cm remote filaments with a Xantopren M Mucosa and Activator NF Optosil (Heraeus, GER) mix for tip coating, first described by (Engel et al., 2011). A 2 cm length of clear i.v. catheter tubing (Portex, Kent UK) was used to aid stability during insertion of the filament.

### 2.3 Surgical preparation

Anaesthesia was induced with 4% isoflurane (Abbott, Berkshire, UK) in room air. Once the animals were unconscious with lack of pedal reflex, they were maintained under 2% isoflurane via a face mask for surgery and, once a tracheal cannula had been inserted, 1% to 1.5% isoflurane for the remainder of the experiment. MCAo was performed by advancing the custom made filament through the external carotid artery (ECA) up to the internal carotid artery to a point just before the MCA branch (Burrows et al., 2015). For imaging experiments, animals were fixed in a stereotactic frame (Narishige, Tokyo, Japan) with ear bars, mouth bar and a dorsal head post to prevent movement. Animals were artificially ventilated with 1% to 1.5% isoflurane in room air via a Zoovent Jetsys ventilator (Universal Lung Ventilators Ltd., Milton Keynes, UK). Body temperature was maintained at 37.5 °C via a heating blanket controlled with a rectal probe (Harvard Apparatus, Kent, UK), and the heart rate was monitored via ECG throughout the experiment. The scalp was dissected down the midline to expose the skull. The bone over the area of the somatosensory cortex supplied by the MCA in the stroked hemisphere was kept translucent with a saline filled paraffin well closed by a circular cover slip.

### 2.4 Two-dimensional optical imaging spectroscopy

**Fig. 1:**
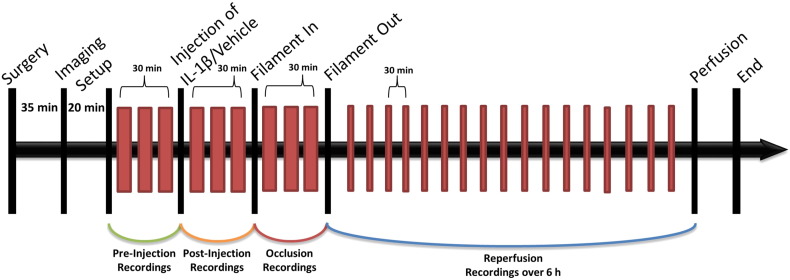
Experimental time line; red bars display 8 minute trials during which imaging data were recorded pre-injection, post-injection, during occlusion and for 6 h following reperfusion. Note that timeline is representative and not to scale.

Two-dimensional spectroscopic imaging data were collected through the imaging window over the intact skull using a high signal-to-noise charged coupled device (CCD) camera (Pantera 1M30, DALSA, Munich, Germany). The region of interest was illuminated sequentially by four different wavelengths of light (550 ± 10 nm, 560 ± 10 nm, 577 ± 10 nm, and 700 ± 10 nm) using a Lambda DG-4 high-speed filter changer (Sutter Instruments, Novato, CA, USA). Camera data collection was synchronized with the filter changer so that each image frame was recorded with one of the four different cortical illumination wavelengths in a sequential manner at a rate of 28 Hz. A ceramic attenuator (PI Instruments, Bedford, UK) was attached to a single whisker on the right whisker pad to enable computer-controlled mechanical stimulation of the barrel cortex throughout the experiment. A single imaging experiment consisted of a continuous recording of 30 trials. Each trial was 16 s long and contained a 4-s pre-stimulus period; 4 s of 8 Hz mechanical whisker stimulation and an 8-s recovery time. These 8-min experiments were recorded before injection, post injection, during, and after MCAo for both experimental groups as shown in Fig. 1.

### 2.5 Imaging protocol

A region of interest (ROI) for imaging was chosen to include the main branches of the MCA and the barrel cortex as identified by localized functional activity from mechanical whisker stimulation. Three sets of trials were recorded for baseline comparison after which the animals underwent i.p. injection with 20 ng/kg IL-1β or vehicle treatment (Fig. 1). Post injection of IL-1β or vehicle, a further three sets of trials were recorded during the 30 min prior to filament advancement. Then the remote filament was advanced ~ 2 to 4 mm to induce a 30 min occlusion of the MCA. During the MCAo three further trials were recorded. The remote filament was then retracted back 6 mm to allow full reperfusion of the MCA. Trials were then recorded every 30 min for 6 h after reperfusion. After 6 h reperfusion, the animal was transcardially perfused with 0.9% saline solution containing 0.5% sodium nitrate followed by 2% paraformaldehyde solution. After fixing, the brain was removed, stored in 2% paraformaldehyde for 24 h and submerged in a 30% sucrose solution for a further 24 h.

### 2.6 Tissue processing

Coronal brain sections (30 μm thick) were cut on a sledge microtome (Leica, Milton Keynes, UK) with freezing stage (Bright Instruments, Huntingdon, UK). Sections were stored in antifreeze solution (30% ethylene glycol and 20% glycerol (Sigma, Gillingham, UK) in phosphate-buffered saline (PBS)) at − 20 °C, before histological staining.

### 2.7 Immunohistochemistry

Brain sections were pre-mounted on charged slides (Fisher Scientific, USA) before antigen-retrieval, where slides were submerged in citrate buffer (Invitrogen) diluted 1:100 in distilled water or 10% methanol (Fisher, Loughborough, UK) with 3% H_2_O_2_ (Sigma, UK) in PBS containing 0.3% Triton X-100 (Sigma) for IL-1 staining. A 1 h blocking step was performed using 2% of normal donkey or rabbit serum (Vector Labs, Peterborough, UK) in primary diluent (PBS containing 0.3% Triton X-100; Sigma) or 2% bovine serum albumin (Sigma) with 5% normal rabbit serum (Vector Labs, UK) for IL-1 staining. Sections were incubated overnight with primary antibodies as follows: mouse monoclonal anti-CD41/integrin alpha 2b antibody (platelets) (1:100, BD, NJ, USA), rabbit anti-Iba-1 (microglia) (1:1000, Abcam) and an immuno double-stain of rabbit anti-Iba-1 (microglia) (1:1000, Abcam) with goat anti-IL-1α (IL-1α co-localisation) (1:100 R&D Systems, USA), primary goat anti-mouse IL-1β IgG antibody (1:500, R&D Systems, Minneapolis, MN, USA). Sections were further incubated with secondary antibodies conjugated to Alexa 488 nm or Alexa 594 nm fluorochromes (1:500, Invitrogen, UK) for 2 h at room temperature or biotinylated rabbit anti-goat antibody (1:500, Vector Labs) for 2 h followed by nickel ammonia diaminobenzidine (DAB) staining procedure (for further details see (Brown et al., 2004, Burrows et al., 2015, Lu and Partridge, 1998, Nielsen et al., 1987) for IL-1 staining. A final wash was performed in PBS before application of Prolong Gold antifade reagent (with or without DAPI; Invitrogen, USA) used as the mounting medium for glass coverslips or DPX mounting medium (Agar Scientific, Stansted, UK). Images for analysis were captured using an Olympus widefield BX51 upright microscope with filter sets matching the fluorescence markers. Equivalent areas of the cerebral cortex at the same magnification (× 20) were imaged across both groups for each specific marker.

Structural images for CD41 platelets and Iba-1 changes were collected on a Leica TCS SP5 AOBS upright confocal microscope. When it was not possible to eliminate cross-talk between channels, the images were collected sequentially. The maximum intensity projections of these 3D stacks are shown in the results. Images were taken at × 40 magnification. IL-1 IHC staining was captured using a brightfield Olympus S2X9 microscope to gain full-slice images.

IgG immunohistochemistry was performed to detect the leakage of endogenous IgG into the brain parenchyma as a measure of BBB breakdown. Sections were washed with PBS followed by incubation with a second blocking agent, consisting of 5% normal horse serum (Vector Labs) and 2% bovine serum albumin in PBS containing 0.3% Triton-X-100 (Sigma) was applied for 1 h. Sections were incubated with biotinylated horse anti-mouse IgG antibody (1:500, Vector Labs). DAB staining protocol, as described above, was performed prior to coverslipping with DPX mounting medium.

Cresyl Violet (CV) stained brain sections were mounted on gelatinized slides and initially soaked in alcohol of increasing concentrations 50%, 70%, 90%, and 100% followed by descending order (4 min each concentration) to remove lipids and fixation chemicals from the tissue. Sections were then submerged with 1.5% CV staining solution. Ultraclear (4 min) was used as a clearing agent, making unstained parts of the tissue transparent followed by coverslipping with DPX mounting medium.

### 2.8 Data analysis

#### 2.8.1 Immunohistochemistry

Images of the cerebral cortex were collected from five evenly spaced sections spanning the somatosensory cortex from both hemispheres for comparison. IgG and IL-1β were quantified by measuring the mean grey value (pixel density) of each hemisphere, the value of which was subtracted from the absolute white value (max), and comparing inter-hemispheric values. Activated microglial (Iba-1) and platelet cells (CD41) were double-blinded to eliminate bias and then counted manually and in each hemisphere in a 0.46 × 0.34 mm size region of the somatosensory cortex. CV cells for cortex counts were centred on layer IV with a 0.3 × 0.3 mm region of interest for all animals.

### 2.9 Calculation of oxyhaemoglobin concentration

**Fig. 2:**
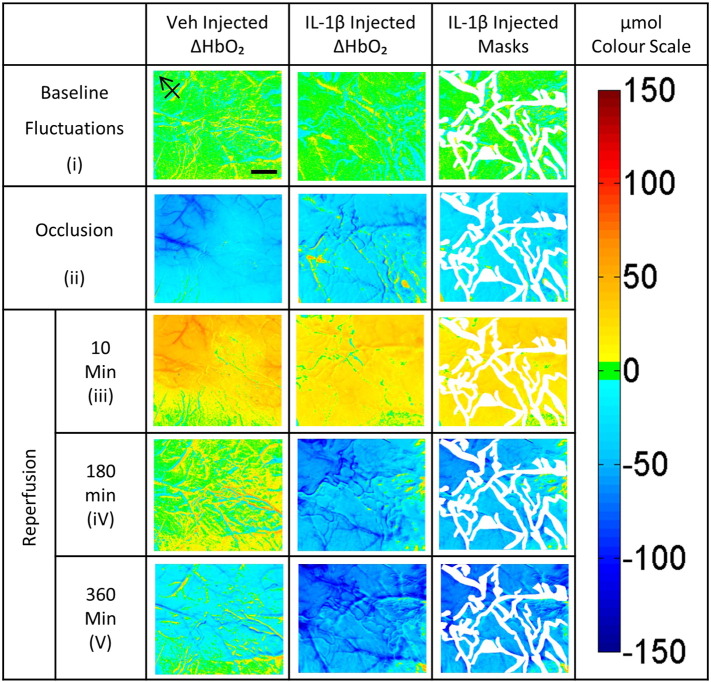
μmolar change in oxyhaemoglobin concentration. The first two columns show typical ∆ HbO_2_ maps for an animal from the vehicle and IL-1 injected groups. The sudden drop in HbO_2_ during occlusion gives instant feedback that a successful occlusion has been initiated. On filament retraction we see an increase in HbO_2_ concentration in both groups. 180 min after removing the occlusion we start to see a decrease of the initial HbO_2_ reperfusion levels for most animals in the vehicle injected group. However the IL-1 injected group shows a significantly worse decrease in HbO_2_. At 360 min post filament removal we have a continued decrease in ∆ HbO_2_ in the vehicle injected group often to levels below those during the ischaemic event. This late reperfusion deficit is even worse in IL-1 injected animals. The third column shows the masks drawn to exclude major vessels from quantitative analysis, leaving only the parenchyma to be quantified and correlated with post-experiment histological processing. The final column represents the μm colour scale. Scale bar = 1 mm, arrow points rostral.

Changes in oxyhaemoglobin concentrations were calculated by spectroscopic analysis with a pathlength scaling Beer Lambert algorithm (Berwick et al., 2005) between the sets of four wavelengths at different time points within the experiment. Baseline variability in cortical HbO_2_ levels was deduced from comparison of an image set recorded before injection with an image set recorded after injection but before occlusion. To establish the extent of the decrease in HbO_2_ during occlusion, we compared the set before occlusion (but after injection) with those at the end of the 30 min occlusion. Once these maps were created, we produced a single mask for each animal to exclude the large arteries and veins from further analysis. This approach allowed the calculation of μmolar changes in ∆ HbO_2_ concentration in the area of microvascular perfusion of the parenchyma before, during and after occlusion, as well as comparisons between groups. For the visualization of ΔHbO_2_ we created a μmol colour scale, with blue for a decrease, red an increase and with green colours indicating little change compared with the reference time point (Fig. 2). For statistical evaluation, the mean pixel values of the regions outside the masks of ΔHbO_2_ maps were calculated. Due to a slight variability of carrying out the complex experiment, the timing within the groups for the data acquisition during early reperfusion ranged from 8 to 12 min after block removal and later reperfusion ranged from 350 to 360 min. The timing of baseline recordings and recordings during occlusion was precise up to the minute (Fig. 2).

### 2.10 Statistical analysis

Animals were randomized for experiments and quantitative analyses were double-blinded across all groups; vehicle group n = 10, IL-1β group n = 10. Past experience and review of referenced publications (Denes et al., 2010a, Denes et al., 2010b, McColl et al., 2007a, McColl et al., 2007b) have shown that a group size of 10 provides reasonable assurance of statistical power for this type of study. One-way ANOVA was used to compare mean HbO_2_ concentration change in μmolar values outside the masked regions in the imaging data and to compare between the two groups for the immunohistochemistry and immunofluorescence marker staining, coupled with Bonferroni multiple comparison test in histology data to indicate differences between the ipsilateral and contralateral hemispheres, calculated and displayed as mean values with error (±) displayed by standard deviation. The nine animals that were sacrificed 70 min after injection were analysed separately and not compared to the animals that had undergone 6 h reperfusion.

## 3 Results

### 3.1 IL-1 treatment increases reperfusion deficit

**Fig. 3:**
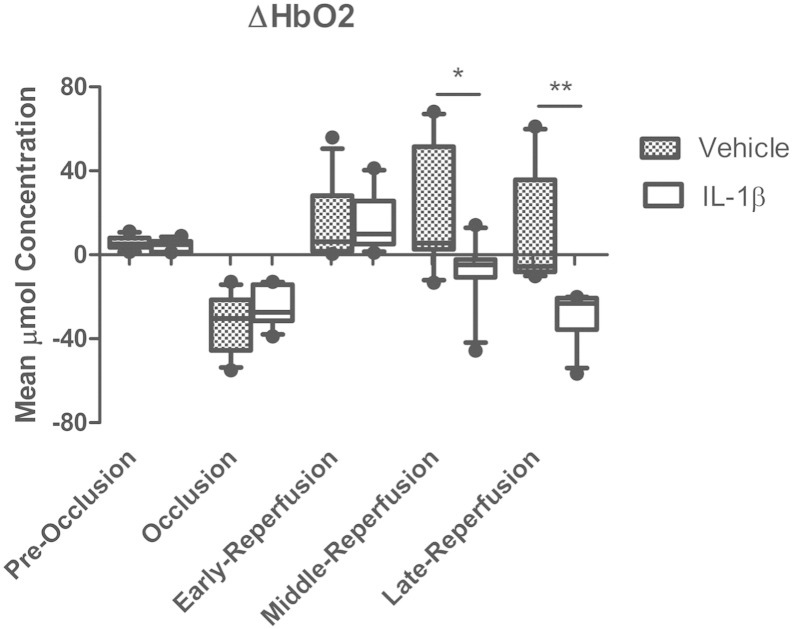
Changes in oxyhaemoglobin. Little variation is observed between the two groups before occlusion for oxyhaemoglobin concentration; 4.35 ± 1.25 μmol IL-1, 5.44 ± 1.52 μmol VEH. Both groups display a significant decrease in HbO_2_ during 30 min MCAo; − 24.99 ± 4.71 μmol IL-1, − 32.01 ± 6.64 μmol VEH. All following changes are calculated with respect to the HbO_2_ value at the end of the occlusion. Initially both groups show an increase in HbO_2_ upon reperfusion; 15.24 ± 6.9 μmol IL-1, 13.63 ± 8.76 μmol VEH. After 180 min of reperfusion (middle reperfusion time point) the IL-1 group HbO_2_ has significantly decreased (− 2.85 ± 10.73 μmol) P < 0.05, compared to the vehicle-injected group (19.06 ± 28.36 μmol). By late reperfusion the HbO_2_ deficit within the IL-1 injected group is more pronounced (− 25.32 ± 8.2 μmol) P < 0.01 compared to the vehicle-injected group (5.64 ± 20.08 μmol) at the same time point. VEH n = 10, IL-1 n = 10.

In order to establish that the i.p. injections themselves do not cause a change of the HbO_2_ concentrations, we compared recordings before and after the injection. Baseline recordings from the two experimental groups show non-significant fluctuations between trials recorded pre-injection (IL-1 or vehicle) and post-injection (∆ HbO_2_ 4.35 ± 1.25 μmol for the IL-1 injected group and 5.44 ± 1.52 μmol for the vehicle injected group) (Fig. 3).

On MCA occlusion there was a 5-to-6-fold decrease in ∆ HbO_2_ in the IL-1 and vehicle injected groups within the stroked hemisphere, with no significant difference between the two treatments (Fig. 3). After 30 min of occlusion, when the filament was retracted, there is an instant 1.6-fold and a 2.3-fold increase in oxyhaemoglobin respectively. ∆ HbO_2_ levels rose to 15.24 ± 6.9 μmol for the IL-1 injected group and 13.63 ± 8.76 for the vehicle injected group. These values display a non-significant variation between the two groups.

At middle reperfusion (180 min) following filament retraction a gradual but significant decline of ∆ HbO_2_ within the brain parenchyma of the IL-1 compared to the vehicle injected group was observed. ∆ HbO_2_ levels decreased 1.3-fold to − 2.85 ± 10.73 μmol (P < 0.05) for the IL-1 injected group compared to a 0.2-fold decrease to 19.06 ± 28.36 μmol for the vehicle injected group (Fig. 3). At late reperfusion (360 min) following filament retraction a continued decline of ∆ HbO_2_ within the brain parenchyma of the IL-1β compared to the vehicle injected group was observed, ∆ HbO_2_ levels decreased 1.6-fold to − 25.32 ± 8.20 μmol (P < 0.01) for the IL-1 injected group compared to a 3-fold increase to 5.64 ± 30.08 μmol for the vehicle injected group, relative to level at the end of the MCAo (Fig. 3).

### 3.2 Increase in biomarkers of ischaemic damage after IL-1 treatment

**Fig. 4:**
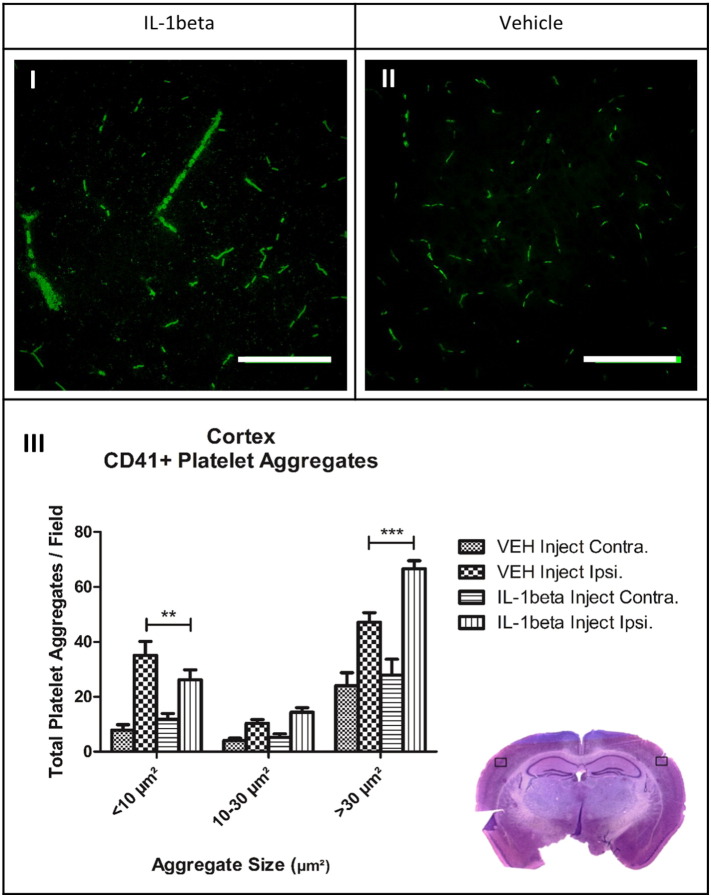
Systemic inflammation causes hyper-coagulation of CD-41 platelets within the ischaemic hemisphere. I) + II) Confocal 2D image projections at × 40 magnification, Larger aggregates were detected within the vasculature of the IL-1 injected mice compared to smaller aggregates observed in the VEH injected group; scale bar displays 50 μm in length. **III)** One-way ANOVA with post hoc Bonferroni multiple comparisons test to compare cell counts of contralateral (contra.) and ipsilateral (ipsi.) hemispheres between the vehicle and IL-1 injected groups; VEH n = 10, IL-1 n = 10. CV stained section displays the region images were taken from.

Hyper-coagulated platelets were observed within the cerebral microvasculature of the stroked hemisphere in IL-1 injected mice compared to vehicle injected (Fig. 4). Platelet clusters were determined by size; small clusters ranged < 10 μm^2^, medium clusters 10–30 μm^2^ and hyper-coagulated clusters > 30 μm^2^, based on the work of (Murray et al., 2013). Systemic injection of IL-1 coupled with 30 min MCAo induced a 48.1% increase of platelet aggregates < 10 μm^2^ (P < 0.01) as well as a 34.3% increase in aggregates > 30 μm^2^ (P < 0.001) within the stroked cerebral cortex compared to the same region within the vehicle injected group (Fig. 4). Morphological changes in platelet aggregation were apparent between the two groups with the IL-1 injected mice displaying large aggregates which fill the diameter and length of larger microvessels. The aggregates themselves appear to show cellular clusters, which could indicate platelet:leukocyte interactions (Fig. 4 (I + II)).

**Fig. 5:**
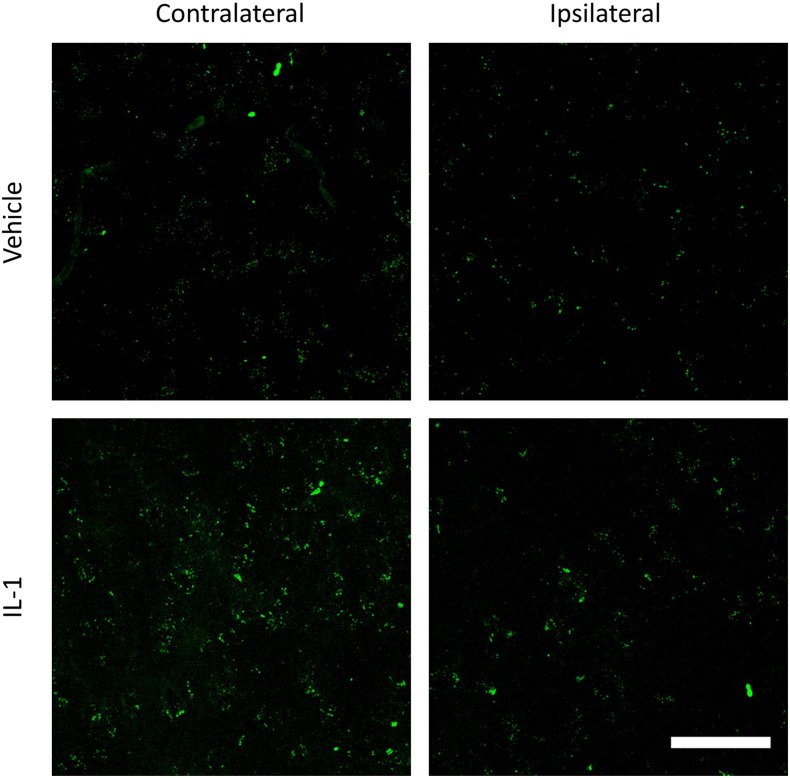
Platelet aggregates 70 min after treatment. Confocal 2D image projections at × 40 magnification. The top row shows an example of cortical tissue in both hemispheres of a vehicle treated animal and the bottom row those after IL-1 injection. Both groups show virtually no large aggregates at this time point; scale bar displays 50 μm in length.

In order to investigate that these platelet aggregates do not appear early before any reperfusion delay we processed the brain tissue that was perfused 70 min after IL-1 (n = 4) or vehicle (n = 5) injection in the same manner. The mean count for any of the three platelet cluster sizes in both the ipsilateral and contralateral hemispheres of both groups was < 3 demonstrating the absence of an early aggregation (Fig. 5).

**Fig. 6:**
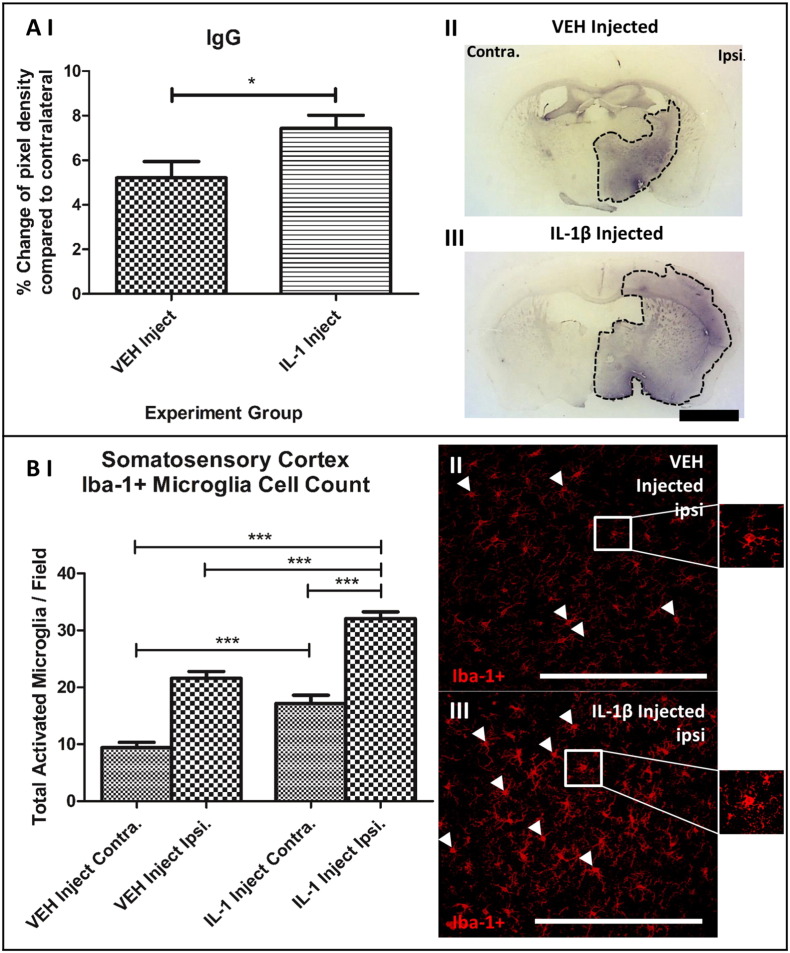
Systemic inflammation causes increased BBB breakdown and exacerbated microglia activation within the ischaemic ipsilateral cerebral cortex. A) + B) One-way ANOVA with post hoc Bonferroni multiple comparison test to compare cell counts of contralateral (contra.) and ipsilateral (ipsi.) hemispheres between the vehicle and IL-1 injected groups. A) II) and III) BBB breakdown was significantly increased in the ipsilateral and contralateral hemispheres of IL-1 injected group compared to the vehicle injected group. Whole slice images are coronal sections from C57/BL6 mice displaying the same point (0.38 mm from bregma) in the brain from a typical vehicle and IL-1β injected mouse. Dotted line demarcates most intense DAB stained regions; scale bar displays 1 mm in length. B II) and III) Cerebral cortex ipsilateral staining for Iba-1 + activated microglia for the vehicle and IL-1 injected groups. Increased activated microglia were observed within the ipsilateral hemisphere of IL-1 injected group compared to the vehicle; scale bar displays 100 μm in length. VEH n = 10, IL-1 n = 10.

In order to determine BBB breakdown leakage of systemic protein IgG into the stroked brain hemisphere was quantified. IgG was localized mainly to the ipsilateral hemisphere and was elevated by 35.3% (P < 0.05) with IL-1 treatment when compared with the vehicle injected group (Fig. 6) Systemic IL-1 injection also increased the number of activated Iba-1 + microglia within the stroked 39.2% (P < 0.001) and contralateral 58.5% (P < 0.001) cerebral cortex compared to the equivalent regions within the vehicle injected group (Fig. 6B). Microglia were considered ‘activated’ upon identification of a bushy rod or amoeboid morphological appearance (Fig. 6B (II + III)).

**Fig. 7:**
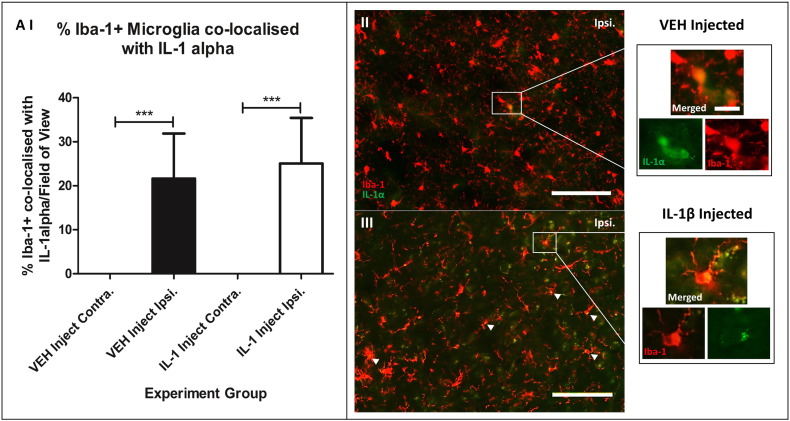
IL-1α was co-localized to Iba-1 + activated microglia in the ipsilateral hemisphere compared the contralateral hemisphere in both groups. I) One-way ANOVA with post hoc Bonferroni multiple comparisons test to compare % of Iba-1 + microglia co-localized with IL-1α per field of view comparing contralateral (contra.) and ipsilateral (ipsi.) hemispheres between the vehicle and IL-1 injected groups. II) + III) × 20 magnification images of cerebral cortex ipsilateral hemisphere double staining of Iba-1 + and IL-1α for vehicle and IL-1β injected groups. Scale bar displays 100 μm in length; VEH n = 10, IL-1 n = 10.

**Fig. 8:**
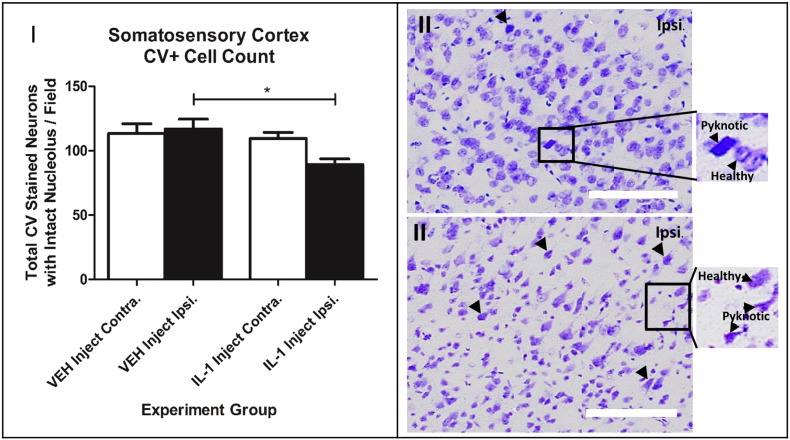
IL-1 decreases healthy CV stained neurons, promoting cell stress and pyknotic morphology compared to vehicle. I) One-way ANOVA with post hoc Bonferroni multiple comparisons test to compare cell counts of contralateral (contra.) and ipsilateral (ipsi.) hemispheres between the vehicle and IL-1 injected groups (only neurons with healthy morphology were counted). II) + III) × 20 magnification images of cerebral cortex ipsilateral hemisphere CV staining for vehicle and IL-1β injected groups. Scale bar displays 100 μm in length; VEH n = 10, IL-1 n = 10.

IL-1α co-localized to Iba-1 stained microglia was not significantly different between the two groups comparing the ipsilateral hemisphere of the vehicle and IL-1β injected group (Fig. 7). Cresyl violet staining revealed a reduction (26.8%, P < 0.05) in the number of healthy neurons in the cortex of the IL-1 treated group, cells being darker stained with angular cell bodies, indicative of pyknotic morphology (Fig. 8).

## 4 Discussion

Our findings show that systemic IL-1 administration just prior to MCAo significantly reduces reperfusion of the cerebral microvasculature when compared to vehicle treated animals, potentially through hyper-coagulation of CD41 + platelets and leukocytes within the cerebral microvessels.

Infection and inflammation-related co-morbidities increase the risk and worsen outcome in stroke patients (Bottcher and Falk, 1999, Grau et al., 1995, Herz et al., 2015, Pinto et al., 2004, Smeeth et al., 2004). IL-1 mimics aspects of infection and has been shown to exacerbate brain damage and neuronal deficit similar to the effects of bacterial endotoxin lipopolysaccharide (LPS) (Denes et al., 2010a, Denes et al., 2010b, Denes et al., 2011, McColl et al., 2007a, McColl et al., 2007b). Furthermore recent data show that infection worsens outcome in experimental stroke via IL-1 dependent mechanisms (Denes et al., 2014). This adds to the already extensive data suggesting IL-1 as a key mediator of ischaemic injury (Murray et al., 2015).The mechanisms by which systemic inflammation exacerbates ischaemic brain damage remain to be fully determined; though effects on the BBB and microvascular reperfusion have all been suggested to have a role (McColl et al., 2007a, McColl et al., 2007b, Murray et al., 2013). Chronic systemic inflammation is associated with increased platelet aggregation after MCA occlusion (Denes et al., 2010a, Denes et al., 2010b) and here we show a direct effect of systemic inflammation on haemodynamic parameters, establishing a pro-coagulant state in which CD41 + platelets hyper-coagulate within the microvasculature in a synergistic response to exacerbated microglial activation. We have also demonstrated that those platelet aggregates do not form early after the ischaemic event, i.e. 10 min after MCAo. Platelet aggregation promotes microvascular dysfunction through activation and release of mediators which promote leukocyte:platelet accumulation (Afshar-Kharghan and Thiagarajan, 2006). Physical obstruction of microvessels compromises collateral blood flow and contributes towards infarct expansion (Del Zoppo et al., 1991). Hyper-coagulated platelet microthrombi could be contributing to the lack of sustained reperfusion within the IL-1 injected group through physical and pathological mechanisms. Platelets bound to the wall of the endothelium become activated, stimulating the release of potent cytokines IL-1α and TNF-α which induce endothelial adhesion molecule upregulation, mediating leukocyte transmigration in to the brain parenchyma (Afshar-Kharghan and Thiagarajan, 2006). Although a definitive role for hyper-coagulated platelets has not been investigated here, in human studies it has been found that a strong systemic platelet activation is associated with thrombin generation, leading to platelet:platelet interactions as opposed to platelet:leukocyte interactions in different subtypes of cerebral ischaemia (Turgut et al., 2011). Experimental studies have found that LPS increases large heteroconjugates of platelets and CD45-positive leukocyte subpopulations (Mirlashari et al., 2002). Systemic IL-1 could be promoting platelet:leukocyte heteroconjugation over platelet:platelet aggregation leading to secondary micro-occlusions within the larger microvessels following an ischaemic challenge. Platelets have also been shown to induce monocyte chemotactic protein (MCP-1) and expression of ICAM-1 in endothelial cells at sites of vascular injury via an IL-1 dependent mechanism (Gawaz et al., 2000). These findings suggest that systemic IL-1 could be exacerbating the coagulation cascade in response to an induced ischaemic event and thus promoting an exaggerated platelet:leukocyte adhesion and coagulation, increasing aggregate size. Confocal images from our immunofluorescence investigations revealed large platelet clusters and cellular morhphological aggregates clogging the lumen and large portions of larger microvessels. Without further markers we were unable to identify if these cellular shapes where in fact leukocytes and further research to specifically delineate the mechanisms by which IL-1β could increase platelet adhesion with other platelets or leukocytes needs to be undertaken.

IL-1α co-localized to Iba-1-positive microglia was more prevalent in the IL-1β injected somatosensory cortex compared to the vehicle injected however these values were not significant. IL-1α activates microvessel endothelial cells to upregulate adhesion molecules; ICAM-1, VCAM-1 and CXCL1, which facilitate transendothelial migration of leukocytes into the brain parenchyma, contributing to BBB breakdown and ischaemic damage (McColl et al., 2007a, McColl et al., 2007b, Thornton et al., 2010). Here we also saw that BBB dysfunction was exacerbated with acute systemic IL-1 administration, agreeing with previous studies where IL-1 was shown to result in loss of tight junctions as a result of matrix metalloproteinase-9 (MMP-9) release from neutrophils, leading to proteolytic breakdown of the BBB (McColl, 2008). Other studies confirm that IL-1 driven leukocyte transmigration results in the release of neurotoxic mediators from neutrophils that can exacerbate neuronal damage (Allen et al., 2012).

In conclusion, we show for the first time that, within 3 h of filament removal, a systemic inflammatory challenge with IL-1 significantly exacerbates reperfusion deficits following a 30 min focal ischaemic event in the brain, highlighting the adverse effects co-morbidity can have on the pathogenesis of ischaemic stroke. These findings re-iterate the importance of including clinically relevant co-morbidities in preclinical stroke research to improve the potential for translational success.

## References

[bb0005] Afshar-Kharghan V., Thiagarajan P. (2006). Leukocyte adhesion and thrombosis. Curr. Opin. Hematol..

[bb0010] Allen C., Thornton P., Denes A., McColl B.W., Pierozynski A., Monestier M., Pinteaux E., Rothwell N.J., Allan S.M. (2012). Neutrophil cerebrovascular transmigration triggers rapid neurotoxicity through release of proteases associated with decondensed DNA. J. Immunol..

[bb0015] Ankolekar S., Rewell S., Howells D.W., Bath. P.M.W. (2012). The influence of stroke risk factors and comorbidities on assessment of stroke therapies in humans and animals. Int. J. Stroke.

[bb0020] Berwick J., Johnston D., Jones M., Martindale J., Redgrave P., McLoughlin N., Schiessl I., Mayhew J.E.W. (2005). Neurovascular coupling investigated with two-dimensional optical imaging spectroscopy in rat whisker barrel cortex. Eur. J. Neurosci..

[bb0025] Bottcher M., Falk E. (1999). Pathology of the coronary arteries in smokers and non-smokers. J. Cardiovasc. Risk.

[bb0030] Brandolini L., Sergi R., Caselli G., Boraschi D., Locati M., Sozzani S., Bertini R. (1997). Interleukin-1 beta primes interleukin-8-stimulated chemotaxis and elastase release in human neutrophils via its type I receptor. Eur. Cytokine Netw..

[bb0035] Brown J.K., Pemberton A.D., Wright S.H., Miller H.R.P. (2004). Primary antibody-Fab fragment complexes: a flexible alternative to traditional direct and indirect immunolabeling techniques. J. Histochem. Cytochem..

[bb0040] Burrows F.E., Bray N., Denes A., Allan S.M., Schiessl I. (2015). Delayed reperfusion deficits after experimental stroke account for increased pathophysiology. J. Cereb. Blood Flow Metab..

[bb0045] Cramer T., Yamanishi Y., Clausen B.E., Forster I., Pawlinski R., Mackman N., Haase V.H., Jaenisch R., Corr M., Nizet V., Firestein G.S., Gerber H.P., Ferrara N., Johnson R.S. (2003). Hif-1 alpha is essential for myeloid cell-mediated inflammation. Cell.

[bb0050] Del Zoppo G.J., Schmid-Schonbein G.W., Mori E., Copeland B.R., Chang C.M. (1991). Polymorphonuclear leukocytes occlude capillaries following middle cerebral artery occlusion and reperfusion in baboons. Stroke.

[bb0055] Denes A., Humphreys N., Lane T.E., Grencis R., Rothwell N. (2010). Chronic systemic infection exacerbates ischemic brain damage via a Ccl5 (regulated on activation, normal T-cell expressed and secreted)-mediated proinflammatory response in mice. J. Neurosci..

[bb0060] Denes A., Thornton P., Rothwell N.J., Allan S.M. (2010). Inflammation and brain injury: acute cerebral ischaemia, peripheral and central inflammation. Brain Behav. Immun..

[bb0065] Denes A., Ferenczi S., Kovacs K.J. (2011). Systemic inflammatory challenges compromise survival after experimental stroke via augmenting brain inflammation, blood–brain barrier damage and brain oedema independently of infarct size. J. Neuroinflammation.

[bb0070] Denes A., Pradillo J.M., Drake C., Sharp A., Warn P., Murray K.N., Rohit B., Dockrell D.H., Chamberlain J., Casbolt H., Francis S., Martinecz B., Nieswandt B., Rothwell N.J., Allan S.M. (2014 May). *Streptococcus pneumoniae* worsens cerebral ischemia via interleukin 1 and platelet glycoprotein Ibα. Ann. Neurol..

[bb0075] Doyle K.P., Simon R.P., Stenzel-Poore M.P. (2008). Mechanisms of ischemic brain damage. Neuropharmacology.

[bb0080] Emsley H.C.A., Smith C.J., Georgiou R.F., Vail A., Hopkins S.J., Rothwell N.J., Tyrrell P.J., I. L-1ra Acute Stoke Investigators (2005). A randomised phase II study of interleukin-1 receptor antagonist in acute stroke patients. J. Neurol. Neurosurg. Psychiatry.

[bb0085] Emsley H.C.A., Smith C.J., Tyrrell P.J., Hopkins S.J. (2008). Inflammation in acute ischemic stroke and its relevance to stroke critical care. Neurocrit. Care..

[bb0090] Engel O., Kolodziej S., Dirnagl U., Prinz V. (2011). Modeling stroke in mice — middle cerebral artery occlusion with the filament model. J. Vis. Exp..

[bb0095] Ericsson A., Liu C., Hart R.P., Sawchenko P.E. (1995). Type-1 interleukin-1 receptor in the rat-brain — distribution, regulation, and relationship to sites of Il-1-induced cellular activation. J. Comp. Neurol..

[bb0100] Gawaz M., Brand K., Dickfeld T., Pogatsa-Murray G., Page S., Bogner C., Koch W., Schomig A., Neumann F.J. (2000). Platelets induce alterations of chemotactic and adhesive properties of endothelial cells mediated through an interleukin-1-dependent mechanism. Implications for atherogenesis. Atherosclerosis.

[bb0105] Grau A.J., Buggle F., Heindl S., Steichenwiehn C., Banerjee T., Maiwald M., Rohlfs M., Suhr H., Fiehn W., Becher H., Hacke W. (1995). Recent infection as a risk factor for cerebrovascular ischemia. Stroke.

[bb0110] Herz J., Sabellek P., Lane T.E., Gunzer M., Hermann D.M., Doeppner T.R. (2015). Role of neutrophils in exacerbation of brain injury after focal cerebral ischemia in hyperlipidemic mice. Stroke.

[bb0115] Lloyd J. (2010). Heart disease and stroke statistics—2009 update: a report from the American Heart Association Statistics Committee and Stroke Statistics Subcommittee (Vol 119, Pg E21, 2009). Circulation.

[bb0120] Lu Q.L., Partridge T.A. (1998). A new blocking method for application of murine monoclonal antibody to mouse tissue sections. J. Histochem. Cytochem..

[bb0125] Maysami S., Haley M.J., Gorenkova N., Krishnan S., McColl B.W., Lawrence C.B. (2015 Aug 4). Prolonged diet-induced obesity in mice modifies the inflammatory response and leads to worse outcome after stroke. J. Neuroinflammation.

[bb0130] McColl B.W. (2008). Systemic inflammation alters the kinetics of cerebrovascular tight junction disruption after experimental stroke in mice. J. Neurosci..

[bb0135] McColl B.W. (2009). Systemic infection, inflammation and acute ischemic stroke. Neuroscience.

[bb0140] McColl B.W., Allan S.M., Rothwell N.J. (2007). Systemic inflammation and stroke: aetiology, pathology and targets for therapy. Biochem. Soc. Trans..

[bb0145] McColl B.W., Rothwell N.J., Allan S.M. (2007). Systemic inflammatory stimulus potentiates the acute phase and Cxc chemokine responses to experimental stroke and exacerbates brain damage via interleukin-1- and neutrophil-dependent mechanisms. J. Neurosci..

[bb0150] McColl B.W., Rose N., FH R., NJ R., CB L. (2010 Feb). Increased brain microvascular MMP-9 and incidence of haemorrhagic transformation in obese mice after experimental stroke. J. Cereb. Blood Flow Metab..

[bb0155] Mirlashari M.R., Hagberg I.A., Lyberg T. (2002). Platelet–platelet and platelet–leukocyte interactions induced by outer membrane vesicles from *N. meningitidis*. Platelets.

[bb0160] Mori E., Delzoppo G.J., Chambers J.D., Copeland B.R., Arfors K.E. (1992). Inhibition of polymorphonuclear leukocyte adherence suppresses no-reflow after focal cerebral-ischemia in baboons. Stroke.

[bb0165] Murray K.N., Buggey H.F., Denes A., Allan S.M. (2013). Systemic immune activation shapes stroke outcome. Mol. Cell. Neurosci..

[bb0170] Murray K.N., Parry-Jones A.R., Allan S.M. (2015). Interleukin-1 and acute brain injury. Front. Cell. Neurosci..

[bb0175] Nielsen B., Borupchristensen P., Erb K., Jensenius J.C., Husby S. (1987). A method for the blocking of endogenous immunoglobulin on frozen tissue-sections in the screening of human hybridoma antibody in culture supernatants. Hybridoma.

[bb0180] Pinto A., Tuttolomondo A., Raimondo D.D., Fernandez P., Licata G. (2004). Cerebrovascular risk factors and clinical classification of strokes. Semin. Vasc. Med..

[bb0185] Rosamond W., Flegal K., Furie K., Go A., Greenlund K., Haase N., Hailpern S.M., Ho M., Howard V., Kissela B., Kittner S., Lloyd-Jones D., McDermott M., Meigs J., Moy C., Nichol G., O'Donnell C., Roger V., Sorlie P., Steinberger J., Thom T., Wilson M., Hong Y., Stroke Amer Heart Assoc Stat Comm (2008). Heart disease and stroke statistics — 2008 update — a report from the American Heart Association Statistics Committee and Stroke Statistics Subcommittee. Circulation.

[bb0190] Semenza G.L. (2009). Regulation of oxygen homeostasis by hypoxia-inducible factor 1. Physiology.

[bb0195] Smeeth L., Thomas S.L., Hall A.J., Hubbard R., Farrington P., Vallance P. (2004). Risk of myocardial infarction and stroke after acute infection or vaccination. N. Engl. J. Med..

[bb0200] Smith C.J., Emsley H.C.A., Gavin C.M., Georgiou R.F., Vail A., Barberan E.M., Zoppo G.J.d., Hallenbeck J.M., Rothwell N.J., Hopkins S.J., Tyrrell P.J. (2004). Peak plasma interleukin-6 and other peripheral markers of inflammation in the first week of ischaemic stroke correlate with brain infarct volume, stroke severity and long-term outcome. BMC Neurol..

[bb0205] Thornton P., McColl B.W., Greenhalgh A., Denes A., Allan S.M., Rothwell N.J. (2010). Platelet interleukin-1 alpha drives cerebrovascular inflammation. Blood.

[bb0210] Turgut B., Turgut N., Celik Y., Tekgunduz E., Pamuk G.E., Demir M. (2011). Differences in platelet–leukocyte aggregates among subtypes of acute cerebral ischemia. J. Neurol. Sci..

[bb0215] Vila N., Castillo J., Davalos A., Chamorro A. (2000). Proinflammatory cytokines and early neurological worsening in ischemic stroke. Stroke.

